# Supplementary material for: The Presence of Micro- and Nanoplastics in Food and the Estimation of the Amount Consumed Depending on Dietary Patterns
Source: Molecules. 2025 Sep 9;30(18):3666. doi: 10.3390/molecules30183666 (PMC12472390; doi:10.3390/molecules30183666)
Supplement: Supplementary file 1 [file molecules-30-03666-s001.zip › molecules-3794422-supplementary.pdf]

molecules

MDPI

Supplementary material

The presence of micro- and nanoplastics in food and the estimation of the amount consumed depending on dietary patterns.

Aleksandra **Duda** and Katarzyna **Petka**

Table S1. Food-based dietary guidelines (FBGDs) for Western European countries - recommended for adult persons' daily intake of products belonging to 7 food groups: (1) water, (2) grains, (3) vegetables and fruits, (4) dairy, (5) protein sources, (6) oils and fats and nuts, and (7) salt and sugar.

Food group	Recommendations
	**Germany** (The nutrition circle - graphical model for the visualization of the dietary guidelines' relative quantities containing 7 segemnts)
1	Daily fluid intake is approximately 1.5 litres. Water is the best choice.
2	The whole-grain varieties of cereal products like bread, pasta, rice and flour are the best choice.
3	At least 5 portions of fruit and vegetables daily, preferably during their harvest season
4	Have some milk and dairy products daily. If plant-based milk alternatives are selected, ensure they are sufficient supply of calcium, vitamin B2 and iodine.
5	Eat legumes at least once a week. Eat fish once to twice a week. Do not consume more than 300 g of meat and sausage per week.
6	Eat a small handful of nuts daily. Choose vegetable oils, rapeseed oils and margarines produced from them, as well as walnut, linseed (flaxseed), soybean and olive oil are also to be recommended.
7	Avoid sweet, salty and fatty foods – they are often "invisibly" present in processed foods like sausage, pastries, sweets, fast food and convenience products.
	**France** (No established food guide, only some recommendations and limitations)
1	No specified.

2	Starchy foods (pasta, bread, rice, semolina, potatoes), which can be eaten every day, by favouring complete starchy foods.
3	Eat at least 5 servings of fruits and vegetables per day.
4	Dairy products (milk, yoghurt, cheese and cottage cheese), 2 per day for adults.
5	Eat legumes (beans, lentils, chickpeas, etc.) at least twice a week.Eat fish twice a week, including one oily fish (sardines, mackerel, herring, salmon). Limited consumption of meats, while favouring poultry and limiting other meats (pork, beef, veal, mutton, lamb, offal) to 500 g per week and the consumption of processed meat, to 150 g per week.
6	Consume a small handful of unsalted nuts per day. Rapeseed, walnut and olive oils (the added fats - oil, butter and margarine - can be consumed every day in small quantities.
7	Limiting the consumption of salty products and salt (to 5 g per day for an adult), as well as sweetened beverages, fatty, sweet, salty and ultra-processed foods.
	**United Kingdom** (the Eatwell Guide – a visual representation of the types and proportions of foods needed for a healthy balanced diet)
1	Drink 6-8 cups/glasses of fluid a day.
2	Base meals on potatoes, bread, rice, pasta or other starchy carbohydrates; choosing wholegrain versions where possible.
3	Eat at least 5 portions of a variety of fruit and vegetables every day.
4	Have some dairy or dairy alternatives (such as soya drinks); choosing lower fat and lower sugar options.
5	Eat some beans, pulses, fish, eggs, meat and other proteins (including 2 portions of fish every week, one of which should be oily).
6	Choose unsaturated oils and spreads and eat in small amounts.
7	Only small amounts of food and drinks that are high in fat and/or sugar.
	**Belgium** (The Épi Alimentaire/Voedingstak, illustrating a spike of grain cereals, represents the top five guidelines associated with the greatest health benefit)
1	Not specified.
2	Whole grain products - At least 125 g per day.
3	Fruits & vegetables - 250g of fruit and at least 300g of vegetables per day. Vary your choices according to seasonal availability.
4	Not specified.

5	Eat legumes at least once a week. For fish, eat fish once or twice a week, focusing on sustainable products high in omega 3 fatty acids. Replace meat with legumes at least once a week
6	Eat 15 to 25 g of plain nuts or seeds (unsalted and/or without a sweet coating) every day.
7	Salt - Limit salt in cooking and do not use salt at the table.
	**Austria** (Food pyramid divided into seven steps, which are further divided into 25 blocks, each representing a daily serving from a food group)
1	Drink at least 1.5 litres of fluid, preferably low-energy drinks in the form of water, mineral water, unsweetened fruit or herbal teas or diluted fruit and vegetable juices. A daily moderate consumption of coffee, black tea (3–4 cups) and other caffeinated beverages is acceptable.
2	Eat 4 servings of grains, breads, pasta, rice or potatoes a day (5 servings for active athletes and children). Prefer wholegrain products.
3	Eat 5 servings of vegetables, legumes and fruits every day. The ideal would be to eat 3 servings of vegetables and/or legumes and 2 servings of fruit. Eat vegetables partly raw and consider seasonal and regional availability when selecting fruits and vegetables.
4	Eat 3 servings of milk and dairy products every day. Prefer low-fat versions.
5	Eat at least 1 or 2 servings of fish (150 g) a week. Prefer high-fat fish such as mackerel, salmon, tuna and herring or local cold water fish such as river trout. Eat up to 3 servings of lean meat or low-fat sausages a week (300–450 g). Eat red meat (such as beef, pork and lamb) and sausages in moderation. Eat up to 3 eggs a week.
6	Consume 1–2 tablespoons of vegetable oils, nuts or seeds daily. High-quality vegetable oils (like olive, canola, walnut, soybean, flax, sesame, corn, sunflower, pumpkin seed and grape seed oil), as well as nuts and seeds, contain valuable fatty acids and can be consumed every day in moderate amounts (1–2 tablespoons). Use baking and frying fats such as butter, margarine or lard and high-fat dairy products such as whipped cream, sour cream and crème fraiche sparingly.
7	Processed foods high in fat, sugar and salt should be consumed sparingly – a maximum of one small serving a day. Try to use herbs and spices instead of salt.

Table S2. Food-based dietary guidelines (FBGDs) for Nordic and Baltic countries - recommended for adult persons' daily intake of products belonging to 7 food groups: (1) water, (2) grains, (3) vegetables and fruits, (4) dairy, (5) protein sources, (6) oils and fats and nuts, and (7) salt and sugar.

Food group	Recommendations
	**Sweden** (Traffic lights - red, yellow, green)
1	Not specified
2	Switch to wholemeal - Choose wholegrain varieties when you eat pasta, bread, grain and rice
3	More vegetables and fruit - Eat lots of fruit, vegetables and berries! Ideally, choose high fibre vegs such as root vegetables, cabbage, cauliflower, broccoli, beans and onions.
4	Switch to low-fat dairy products
5	More seafood - Eat fish and shellfish two to three times a week. Vary your intake of fatty and low-fat varieties, and choose ecolabelled seafood.<br>Less red and processed meat - Eat less red and processed meat, no more than 500 grams a week. Only a small amount of this should be processed meat.
6	Switch to healthier fat. Choose healthy oils when cooking, such as rapeseed oil or liquid fats made from rapeseed oil, and healthy sandwich spreads.
7	Less salt - Choose food with less salt. Use less salt when you cook, but choose salt with iodine when you do use it.<br>Less sugar - Hold back on the sweets, pastries, ice creams and other products containing lots of sugar. Cut back on sweet drinks in particular.
	**Finland** (a food triangle and a food plate model)
1	Drink water when you are thirsty. Decrease consumption of soft drinks and sweet juices.
2	Eat wholegrain cereals (bread, porridge, pasta, etc.) several times a day. Prefer fibre-rich and low-salt products. Avoid products made of refined flour with plenty of hard fat and sugar.
3	Eat vegetables, fruits and berries frequently (a minimum of 500 g/day, excluding potatoes.
4	Consume fat-free/low-fat milk products daily (5–6 dl/day) and two or three slices of low-fat cheese.
5	Eat fish (of different kinds) two to three times a week. When eating meat, choose low-fat, low-salt products and limit the amount of red meat and meat products to < 500 g a week.
6	Use soft vegetable oil based spreads on bread and vegetable oils in cooking and salads.

7	Use low-salt products (salt intake should be < 5 g/day).
**Norway** (No a food guide, only some messages to consumers)	
1	Choose water as a thirst quencher.
2	Eat wholegrain products every day.
3	Eat at least 5 portions of vegetables, fruits and berries each day.
4	Include lean dairy products as part of your daily diet.
5	Eat fish for dinner two to three times a week. Fish is also a great filling in sandwiches. Choose lean meat and lean meat products. Limit the amount of processed meat and red meat you consume.
6	Not specified.
7	Choose foods with a low salt content and limit the use of salt when preparing food. Limit your consumption of food and drink with a high sugar content.
**Iceland** (a food circle divided into six food groups: fruits and vegetables, cereals and cereal products, dairy products, animal source foods and nuts and lastly oils and visible fats and water is at the centre of the circle)	
1	It is recommended to drink tap water when thirsty, with meals, and while exercising.
2	Whole grain products - preferably 3-4 servings a day (equivalent to 90 grams daily).
3	Fruits and a lot of vegetables (5 portions a day, at least half of it should be vegetables, fruit juice not included).
4	Low fat dairy products without sugar (2 portions a day) = Drinking or eating 350 to 500 millilitres or grams of milk or dairy products per day is recommended. Choose low-fat, non-sweetened dairy products or cultured milk.
5	Fish 2 to 3 times a week (desirable to have one of the fish meal fatty fish) = Eating 300–450 grams of fish per week is recommended, which means a cooked quantity, ready to eat. At least 200 grams should be of fatty fish. Meat in moderation (limit the consumption of red meat to 350 grams per week, especially limit the consumption of processed meat). A moderate consumption of eggs.
6	Increase the proportion of soft fats (unsaturated fats) in the diet, which come from vegetable oils, fatty fish, fish oil, nuts, and seeds. Eating 20-30 grams of unsalted nuts per day is recommended.
7	Salt intake should not exceed 6 grams per day. Limit or avoid drinks with sugar or non-sugar sweeteners, such as soft drinks, energy drinks, iced tea, and juice.

**Denmark** (food guide are presented by poster with 6 boxes representing different food groups with the size of each proportional to the participation in daily consumption).	
1	Drinking 1-1½ liters of liquids per day. Drink water. If you drink coffee as an adult, then drink no more than 4 cups a day. Drink at most 100 mL of juice per day.
2	Eat wholegrain foods. Eat 90 g of wholegrain a day or more. For example, 90 g of wholegrain corresponds to a portion of oatmeal, one slice of wholegrain rye bread and a small portion of wholegrain pasta. It is important to eat different types of wholegrain products. Choose wholegrain varieties when you eat bread, pasta, rice, crispbread, breakfast cereals and porridge and when you, for example, buy sandwiches or takeaway. Include potatoes in your meals several times a week. About 100 g of potatoes a day is adequate when eating a plant-rich and varied diet.
3	Eat 600 g of vegetables and fruit a day – that is, '6 a day'. At least half should be vegetables. Eat different types: about 100 g of vegetables or fruit is for example equivalent to a large carrot or an apple. Dark green vegetables such as spinach, broccoli and kale (about 100 g a day is adequate). Red and orange vegetables such as carrots, tomatoes and red peppers (about 100 g a day is adequate) Choose seasonal vegetables, fruits and berries. Small glass of juice (100 ml) can count as 1 of your '6 a day', but only as 1.
4	Choose mainly skimmed milk or buttermilk. Choose mainly fermented milk products, such as plain yogurt, with a maximum of 1.5% fat and choose mainly cheese with a maximum of 17% fat (30+). Limit your intake of high-fat dairy products, such as cream. 250-350 ml milk or milk products a day is adequate when eating a plant-rich and varied diet. The quantity depends on your age (see the info box on the right). Also, use cheese in your food or in sandwiches. About 20 g of cheese (1 slice) a day is adequate when eating a plant-rich and varied diet. The quantity is less for children.
5	Eat less meat – choose legumes and fish Cut down on meat. About 350 g of meat a week is adequate when eating a plant-rich and varied diet. Limit especially beef and lamb. Limit processed meat, such as smoked and salted meat, as much as possible. Choose mainly meat and meat products with a maximum of 10 % fat. Eat 350 g of fish per week, of which 200 g is oily fish. Oily fish include for example, herring, mackerel, salmon and trout. It is important to eat different types of fish. All kinds of fish, shellfish and roe count in the 350 g. Eat more legumes, such as brown, white and black beans, kidney beans, lentils and chickpeas. About 100 g a day (in addition to the 600 g of vegetables and fruits) is adequate when eating a plant-rich and varied diet. Eat about 30 g of nuts a day. Vary between different types, such as walnuts, hazelnuts and almonds. Choose unsalted nuts or nuts with a maximum of 0.8 g of salt per 100 g. Supplement your meals with seeds. About 1-2 tablespoons of seeds a day is adequate when eating a plant-rich and varied diet. Choose for example, sesame seeds, pine nuts and pumpkin seeds. Bread can be a good source of seeds. For variation in your meals, eat eggs. About 3 eggs a week is adequate when eating a plant-rich and varied diet.
6	For cooking, choose vegetable oils like rapeseed and olive oil rather than solid fats, such as butter and coconut oil. Limit the use of butter on bread and sandwiches. Choose for example hummus or a little pesto instead.
7	Eat less sweet, salty and fatty food. A maximum of 5 handfuls a week is a guideline for how much snacks and sweets you have room for in your diet when you eat healthy and varied.

**Latvia** (Food guide is represented by a plate displaying food groups: ½ fruits, vegetables, greens and berries; ¼ grains and potatoes; ¼ protein products (meat, eggs, fish, dairy, legumes, nuts and seeds) and small portions fats and oils plus liquids)	
1	Drink 1.5–2 litres of liquid, including water, every day.
2	Eat 4-6 servings of cereals, cereal products (preferably wholegrain) and potatoes every day.
3	Eat at least 5 servings of vegetables, fruits and berries every day. Try to choose local and fresh products.
4	Eat 2-3 servings of dairy products every day. Prefer low-fat versions of milk and dairy products.
5	Eat legumes, fish or lean meat. The recommended daily amount of those products is 2-3 servings. Eat fish at least twice a week.
6	Reduce your consumption of margarine, butter and fatty meats. Use little oil to prepare your meals.
7	Limit consumption of salt and sugar and products containing them.

Table S3. Food-based dietary guidelines (FBGDs) for Central and Eastern European countries - recommended for adult persons' daily intake of products belonging to 7 food groups: (1) water, (2) grains, (3) vegetables and fruits, (4) dairy, (5) protein sources, (6) oils and fats and nuts, and (7) salt and sugar.

Food group	Recommendations
	**Bulgaria** (Food pyramid of 6 food categories)
1	Drink plenty of water every day.
2	Consume cereals as an important source of energy. Prefer wholegrain bread and other wholegrain products.
3	Eat a variety of vegetables and fruits more than 400 grams every day, preferably raw
4	Prefer milk and dairy products with low fat and salt content.
5	Choose lean meat, replace meat and meat products often with fish, poultry or legumes.
6	Limit total fat intake, especially animal fat. Replace animal fats with vegetable oils when cooking.
7	Limit the consumption of sugar, sweets and confectionery, avoid sugar-containing soft drinks. Reduce intake of salt and salty foods.
	**Poland** (plate with the recommended proportions of food groups or Healthy Lifestyle Pyramide)
1	Replace sweetened beverages with water.
2	Eat more whole-grain cereal products (e.g. oatmeal, wholemeal bread, wholemeal pasta, groats). Replace processed cereal products (e.g. white bread, sweet breakfast cereals) with whole-grain cereal products.
3	Eat more various, colorful vegetables and fruits – more vegetables than fruits.
4	Eat more low-fat dairy products (in particular fermented). Replace full-fat dairy products with low-fat dairy products (milk, yogurt, kefir, buttermilk, and white cheese).
5	Eat more legume seeds (e.g. beans, peas, chickpeas, lentils, broad beans), fish (in particular fatty sea fish). Eat less red meat and processed meat (sausages, bacon). Replace it with fish, poultry, eggs, legume seeds and nuts.
6	Eat more nuts and seeds (e.g. walnuts, pumpkin seeds, sunflower seeds). Replace animal fats with vegetable oils.
7	Eat less: salt, sugar and sweetened beverages processed food products (such as fast food, salty snacks, biscuits, bars) high in salt, sugars and fats.
	**Hungary** (a plate of food)

1	8 glasses of fluids daily, with at least 5 glasses being water.
2	3 portions of grains (with at least one portion whole grain).
3	At least 500g of vegetables or fruit daily. Fruit and vegetables should be a half of the daily food portion.
4	500 ml of milk or dairy products.
5	Eat a wide variety of protein sources, with limited consumption of red meat (500-700 g per week) and processed meat.
6	Add as fat as possible in your cooking.
7	Add as little salt and sugar as possible in your cooking.
**Romania** (food pyramid divided into seven food group)	
1	2-2,5 L water.
2	Eat plenty of cereals (6-11 servings).
3	Eat plenty of fruits and vegetables.2-4 serving of fresh fruits.
4	2-3 servings of dairy products.
5	3-4 servings of legumes and 2-3 dishes with lean meat (white meat).
6	Choose foods that contain small amounts of fat.
7	Eat highly processed foods high in sugar sparingly.
**Czech Republik** (no graphical presentation of 13 rules “Zdravá třináctka”)	
1	Drink at least 1.5 litres of fluids (water, low or medium mineral content still mineral water, weak tea, fruit teas and juices, preferably unsweetened or diluted) every day.
2	Eat whole grain products and don't forget about legumes (at least once a week).
3	Eat enough vegetables (raw and cooked) and fruit, at least 400 g per day (twice as much vegetables as fruit), divided into several portions; don't forget to eat small amounts of nuts.
4	Consume milk and dairy products every day, especially fermented ones (e.g. yoghurt, fermented milk drinks, kefir); choose products with a medium fat content.
5	Eat fish and fish products at least twice a week.

6	Control your fat intake, limit the amount of hidden fat (fatty meat, fatty meat and dairy products, delicate and long-life baked goods with a higher fat content, crisps, chocolate products) and when preparing meals. Prefer fats low in saturated fatty acids.
7	Limit your sugar intake, especially in the form of sweetened drinks, sweets, jams, sweetened dairy products and ice cream. 10. Limit your intake of table salt and products with a high salt content (cold cuts, fish products, cheese, crisps, salted sticks and nuts), and do not add salt to ready-made meals.
	**Slovak** (a healthy plate)
1	Drink at least 2 liters of liquids per day, prefer water and drink with non-added sugar.
2	To increase your fiber intake you could: go for whole grains breads, pastries or wholemeal.
3	Consume at least 5 portions of fruit and vegetables each day.
4	Increase intake of skimmed milk, low fat yoghurt and reduced fat cheese.
5	Choose a variety of proteins, which include fish, lower-fat meat and poultry, eggs, beans and peas, soya products and natural seeds and unsalted mixed nuts.
6	Replace saturated fats (e.g. margarine) by virgin oils (e.g. sunflower seeds oil, olive oil).
7	Reduce intake of salt to less than 5 g of salt per day. Avoid consuming a lot of foods or drinks with added sugar.

Table S4. Food-based dietary guidelines (FBGDs) for Southern European countries - recommended for adult persons' daily intake of products belonging to 7 food groups: (1) water, (2) grains, (3) vegetables and fruits, (4) dairy, (5) protein sources, (6) oils and fats and nuts, and (7) salt and sugar.

Food group	Recommendations
	**Italy** (no developed graphical representation of food guidelines)
1	Drink abundant water every day.
2	Eat whole grain and legumes.
3	Eat more fruits and vegetables.
4	No specified.
5	Not specified but it is recommended to select poultry or legumes over red meat and small fish from the Mediterranean sea over exploited species.
6	Fats: select which ones and limit the quantity.
7	Sugar, sweets and sugar sweetened beverages: less is better. Salt: less is better (but iodised).
	**Greece** (Pyramid which is in line with the traditional Mediterranean diet, food product divided into groups of daily consumption (wholegrain cereals and products, fruits, vegetables, olive oil and dairy products), weekly (fish, poultry, olives, pulses, nuts, potatoes, eggs and sweets) and monthly (red meat)
1	Drink plenty of water.
2	Consume a variety of cereals every day. Prefer non-refined cereals and products (whole grain bread and pasta, brown rice etc.).
3	Consume a variety of fruit and vegetables every day.
4	Prefer low-fat dairy products.
5	Limit red meat consumption. Choose lean cuts. Avoid processed meat. Consume fish and seafood frequently. Choose small fatty fish. Consume legumes frequently.
6	Use olive oil as the main added fat.
7	Limit salt and added sugar intake.
	**Spain** (Healthy eating plate which consists of 50% fruits and vegetables, 25% whole grains, and 25% healthy protein).
1	Water: Water is the drink of choice for a healthy diet. Drink water whenever you are thirsty. Preferably tap water

2	Cereals: Between 3 and 6 servings a day, depending on whether you lead a more or less active life. A moderate intake of potatoes and other tubers (baked or boiled is preferred). Prioritising whole grains is recommended. Eat different types of cereals to encourage crop diversity.
3	Vegetables: At least 3 servings or 150-200g per day. Buy them fresh and local. Fruits: At least 2-3 servings or 120-200g per day.
4	Up to 3 servings per day of milk and dairy products, preferably without added sugars and with low salt content. Reduce the number of daily servings of dairy products if other foods of animal origin are consumed.
5	Legumes, at least 4 servings a week up to a daily consumption. Fish, at least 3 servings a week, prioritising blue fish and species with less environmental impact. Eggs, maximum of 4 medium-sized per week. A maximum of 3 servings/week of meat, prioritising poultry and rabbit meat and minimising the consumption of processed meat.
6	Nuts, consume 3 or more servings a week (up to 1 serving/day). Use olive oil in all your meals, as a dressing and in food preparation. Reduce or even avoid butter and other saturated fats.
7	Reduce or even avoid salt, sugar, sugary and sweetened beverages and processed foods high in sugars, fats and salt.
**Turkey** (Four-leaf clover divided into four basic food groups: milk and dairy; meat, eggs, fish, legumes and seeds; vegetables and fruit; and bread and cereal	
1	Drink plenty of fluids. Drink much water and sugar free herbal teas. Instead of sugar added soft drinks please prefer skim milk, ayran (watered yogurt) and kefir.
2	Increase consumption of wholegrain cereals and leguminous seeds. Eat whole cereal products in appropriate amount everyday even every meal.
3	Vegetable or fruit must be consumed at least 5 servings daily. At least two servings of vegetable and fruit must be vegetables with green leaves or citrus, fruits like oranges or tomato,
4	Adults should consume at least 500 grams of milk or yogurt (2 servings) – preferably no fat or low fat and cheese with low salt.
5	Daily 2 servings from the group of meat-eggleguminous seeds should be consumed . Fish must be eaten twice a week for healthy nutrition because it contains omega-3 (n-3) fatty acid. Meats must be consumed in limited amounts because of their saturated fat content. Since its protein quality is high, an egg must be consumed daily by children.
6	Decrease the consumption of saturated fat (butter, margarine, animal fat).
7	Avoid taking much salt and sugar.
**Portugal** (A wheel divided on 7 parts illustrating major food groups – based on Mediterranean diet)	
1	Prefer Water to Beverages Containing Added Sugar, Alcohol, and Caffeine.
2	Potato, Cereal, and Cereal Products: A significant part of the diet, with 4 to 11 portions recommended each day.

3	At t least 400 g of fruits and vegetables daily. Vegetables - recommendation of 3 to 5 portions daily. And fruits - advised at 3 to 5 portions per day.
4	Milk and Dairy Products: Advised to consume 2 to 3 portions daily.
5	Meat, Fish, Seafood, and Eggs: Suggested intake ranges from 1.5 to 4.5 portions per day. Pulses: It is recommended to have 1 to 2 portions daily.
6	Fats and Oils: Recommended daily intake of 1 to 3 portions.
7	Limit Consumption of Products with High Sugar Content to Special Occasions. Limit Consumption of Salt to Less than 5g a Day. Moderate Consumption of Foods and Food Products High in Salt Such as Cold Meats, Canned Foods, Chips, and Salty Snacks.
	**Albania**
1	Take 1-2 liter of beverages per day choosing those with no sugar added but potable water, mineral water and teas with little sugar added.
2	Take bread, grains, rice or potatoes. The regular consumption of the grains is the basis for healthy consumption. Take a plate of grains every main meal (i.e. 3 portions per day, 1 portion= 75-125 g bread or 60-100 g legumes like lentils, kidney beans, chick-peas, etc. or 180-300 g potatoes, or 45-75 g of pasta or rice, etc.) At least two portions should be integral.
3	Various kinds of vegetables and fruits many times per day (at least 400 g/day divided into 5-6 portions) possibly fresh and locally produced. Take everyday 3 portions of vegetables (1 portion = minimum120 g vegetables salad or soup). Take every day 2 portions of fruits (1 portion = minimum 120 g). 1 portion of fruits or vegetables per day may be substituted with 200 ml juicy fruit or vegetable juice with no sugar added.
4	Use milk and its by-products (sour cream, yoghurt, cheese, etc. which have low rates of fat and salt. Take 3 portions of milk or other by-products like yoghurt, curdle cheese with the lowest percentage of fat (1 portion = 200 ml milk, or 150-180 g yoghurt, or 60-90 g curdle cheese).
5	Substitute greasy meat and meat by-products with peas, kidney beans, lentils, fish, poultry, or beef. Take everyday alternatively one portion of meat or fish, an egg or a portion of cheese (1 portion = 100-120 g meat or processed fish, or 2-3 eggs, or 200 g fresh cheese, or 50-60 g ripened cheese).
6	Control the consumption of fat (no more than 30 % of the daily energy) and substitute saturated fat with light vegetal oils such as light margarines. Use mostly 2 tea spoons (10-15 g) high quality vegetal oil/day (mainly olive oil) for salads. Use 2 -3 tea spoons (10-15 g) vegetal oil for cooking and preparing processed food (fry, stew, bake, etc) e.g. sunflower oil, peanut oil, corn oil, etc. Use 2-3 tea spoons (10 g) of butter or vegetal margarine which has very high nutritive. It is recommended to take a portion of fatty fruits per day (1 portion = 20-30 g almonds, hazelnut or nut, etc.).
7	Use iodine fortified salt with limited portions (less than 5 g salt per day). Choose food containing less sugar and do not use too much sugar, reducing the sweet beverages and deserts.
	**Croatia** (Food pyramid divided into four levels)

1	Not specified.
2	Eat plenty of wholegrain cereals.
3	Eat five or more servings of fruits and vegetables a day.
4	Not specified.
5	Choose lean meats (e.g. poultry, rabbit) and fish over red meat.
6	Include in your daily diet high-quality vegetable oils, such as olive and pumpkin seed oil.
7	Limit your intake of sweets; replace them with nuts and dried fruits. Limit your intake of fat and salt. Snacks should be chosen carefully – give preference to fresh and dried fruits, nuts and fermented milk products.

Based on: Piramida - Food and Nutrition Institute Available online: https://ncez.pzh.gov.pl/wp-2356 content/uploads/2021/03/piramidaizz_dorosli.pdf (accessed on 5 July 2025) [355], Europe, W.H.O.R.O. for Food-Based Dietary Guidelines in the WHO European Region. 2003 [362] and FDGB FAO Available online: http://www.fao.org/nutrition/education/dietary-guidelines/regions/countries/en/ (accessed on 20 August 2025) [363].
